# Magnetically Driven Hydrogel Surfaces for Modulating Macrophage Behavior

**DOI:** 10.1021/acsbiomaterials.4c01624

**Published:** 2024-10-09

**Authors:** Lanhui Li, Els Alsema, Nick R. M. Beijer, Burcu Gumuscu

**Affiliations:** † Biosensors and Devices Lab, Institute for Complex Molecular Systems, Eindhoven University of Technology, 5600MB Eindhoven, The Netherlands; ‡ Biointerface Science Group, Institute for Complex Molecular Systems, Eindhoven University of Technology, Eindhoven, 5600MB Eindhoven, The Netherlands; § Centre for Health Protection, National Institute for Public Health and the Environment (RIVM), 3720BA Bilthoven, The Netherlands

**Keywords:** magnetic hydrogels, dynamic stiffness change, pulsed magnetic field, modulated macrophage polarization

## Abstract

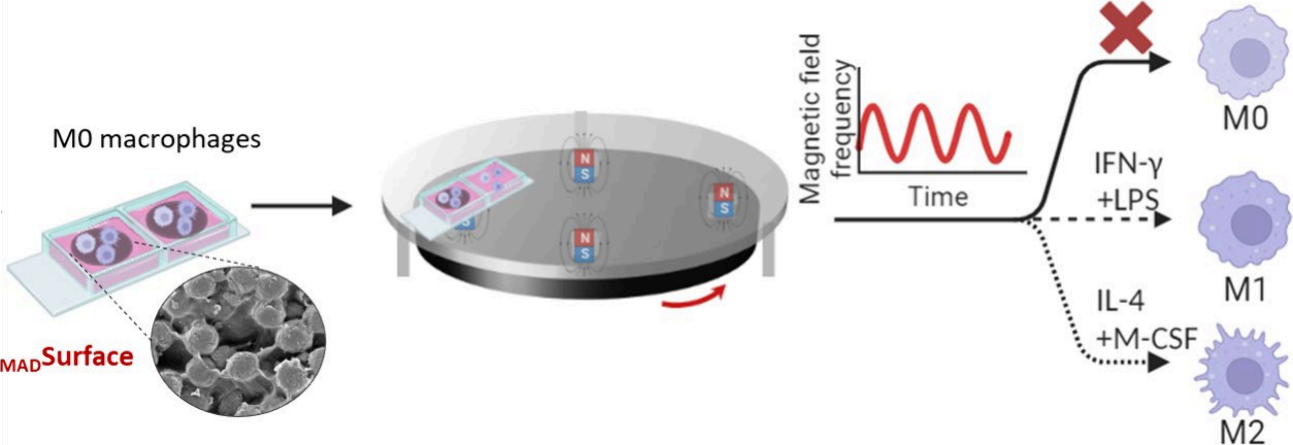

During the host response toward implanted biomaterials, macrophages can shift phenotypes rapidly upon changes in their microenvironment within the host tissue. Exploration of this phenomenon can benefit significantly from the development of adequate tools. Creating cell microenvironment alterations on classical hydrogel substrates presents challenges, particularly when integrating them with cell cultivation and monitoring processes. However, having the capability to dynamically manipulate the cell microenvironment on biomaterial surfaces holds significant potential. We introduce magnetically actuated hydrogels (_Mad_Surface) tailored to induce reversible stiffness changes on polyacrylamide hydrogel substrates with embedded magnetic microparticles in a time-controllable manner. Our investigation focused on exploring the potential of magnetic fields and _Mad_Surfaces in dynamically modulating macrophage behavior in a programmable manner. We achieved a consistent modulation by subjecting the _Mad_Surface to a pulsed magnetic field with a frequency of 0.1 Hz and a magnetic field flux density of 50 mT and analyzed exposed cells using flow cytometry and ELISA. At the single-cell level, we identified a subpopulation for which the dynamic stiffness conditions in conjunction with the pulsed magnetic field increased the expression of CD206 in M1-activated THP-1 cells, indicating a consistent shift toward the M2 anti-inflammatory phenotype on _Mad_Surface. At the population level, this effect was mostly hindered in the culture period utilized in this work. The _Mad_Surface approach advances our understanding of the interplay between magnetic field, cell microenvironment alterations, and macrophage behavior.

## 1 Introduction

Mechanical forces play a pivotal role in various cellular processes, including cell differentiation and behavior changes. Here, mechanotransduction determines how cells react to, transmit, and convert these mechanical cues into chemical signals within the cells.^1,2^ Unraveling the underlying molecular mechanisms governing cellular responses to mechanical forces holds the potential for influencing cellular behavior, possibly with a connection to in vivo treatment techniques.^3^ A fundamental requirement in investigating cellular mechanotransduction involves the precise and reversible application of time-controlled mechanical forces across a cell culture.^4^ This allows for the evaluation of the cell behavior over time upon changing the mechanical loading. The time-controlled mechanotransduction is particularly interesting for macrophages. Intermittent control of mechanotransduction emerges as a promising strategy to modulate immune cells like macrophages in changing their cytokine secretion profile from pro-inflammatory (M1) toward a more anti-inflammatory and regenerative (M2) profile in the process of wound healing after surgery.^5^ The shift in the cytokine secretion profile in macrophages can be controlled by the mechanical properties of their microenvironment.^6,7^

Physical alterations have been implemented *in vitro* within the cellular microenvironment through various methods including atomic force microscopy,^8^ optical approaches,^9,10^ cell stretching,^11^ manipulation of microposts,^12^ and the utilization of microfluidic chips,^13^ targeting both individual cells and bulk configurations. Additionally, diverse biomaterials with varying compositions have been employed to modify surface topography,^14,15^ stiffness,^16^ and composition.^17^ These modifications aim to guide macrophages toward secreting anti-inflammatory markers, ultimately striving for enhanced tissue healing outcomes.^18^ In all cases, such approaches are unable to achieve high spatial and temporal resolution in mechanical stimulation. The application of magnetic fields has been explored to achieve both challenges with a hybrid approach. Magnetic fields offer the ability to reversibly and dynamically impact the stiffness and morphology of magnetic substrates,^19^ enabling precise manipulation of the exposed cells and their created microenvironment such as their extracellular matrix.^20^ In the context of immune cell polarization, magnetic fields have been used in microparticle formats that change stiffness and shape^21,22^ as well as nanoparticle formats.^23,24^ Previous studies expanded our knowledge of the magnetic particle–macrophage behavior relationship; yet, only a few studies compared the cell behavior in individual cells and bulk configurations. This aspect is important in fundamental studies where immune cell population heterogeneity is investigated.

In this work, we introduce magnetically actuated dynamic polyacrylamide hydrogel surfaces (_Mad_Surface) to modulate changes in the cytokine expression profiles in macrophages. This is a hybrid approach that brings the dynamicity and reversibility of magnetic field application to the cell culture microenvironment. The basis of the _Mad_Surface consisted of embedded magnetic microparticles in a polyacrylamide (PAM) hydrogel scaffold, which was subjected to a pulsed magnetic field upon the exposure of a set of permanent magnets fixed on a rotating stage. We cultured THP-1 cells on this platform with a continuously applied pulsed magnetic field for 24 h while applying a differentiation protocol to obtain M0 naïve macrophages and M1 pro-inflammatory and M2 anti-inflammatory phenotypes. We observed that the heterogeneity in the cell population grows upon exposure to the pulsed magnetic field and stiffness changes within 24 h. This shows the potential of this approach to provide controlled environments for studying immune responses and further advance the knowledge of the relationship between dynamic surface mechanics and macrophage behavior.

## 2 Results and Discussion

### 2.1 Characterization of Hydrogel Surfaces, Pulsed Magnetic Field, and Cell Attachment

**Figure 1:**
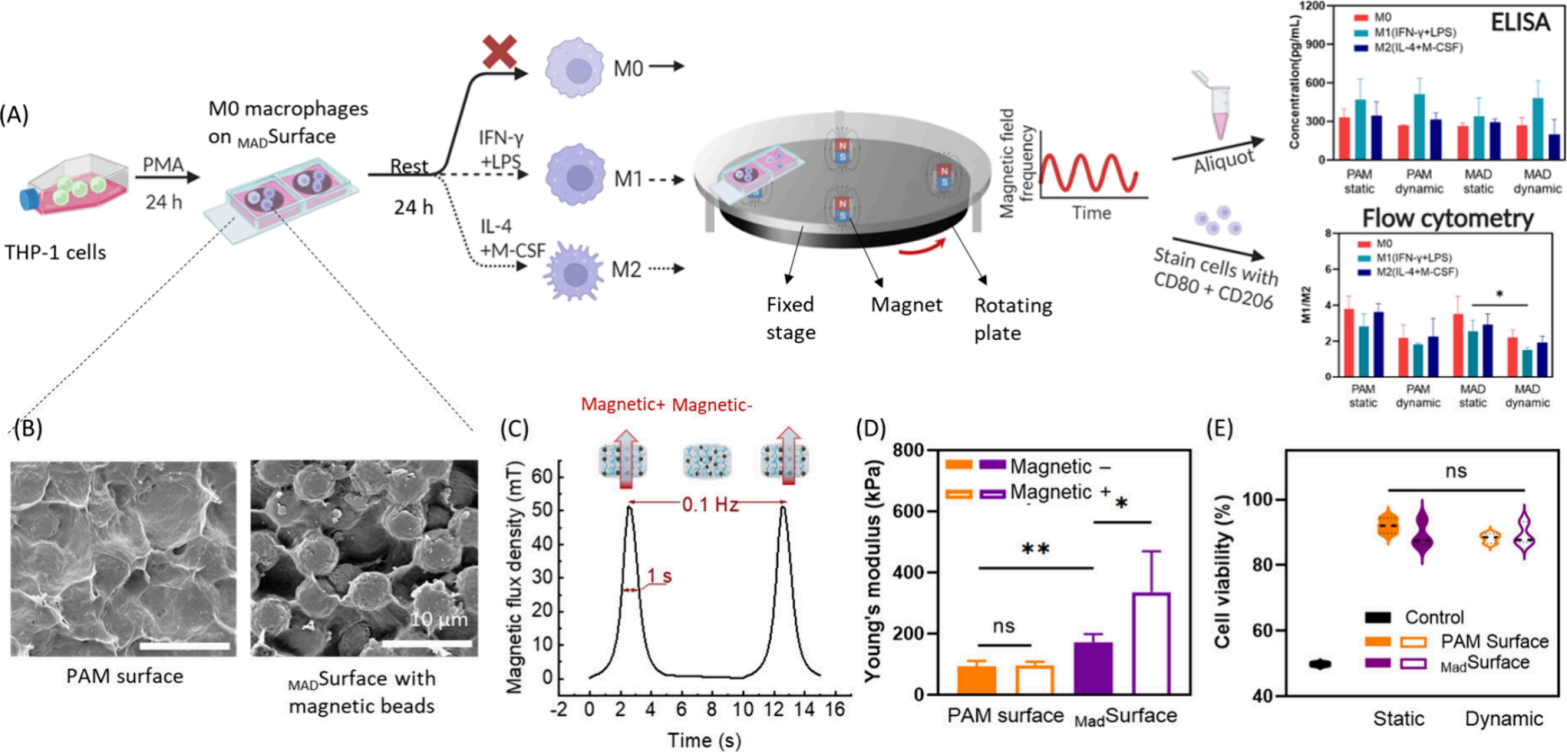
Fabrication, characterization, and application of _Mad_Surface for changing macrophage behavior. (A) Schematic view of the process flow of differentiation THP-1 cells into M0 macrophages, seeding the cells on _Mad_Surface, and application of a pulsed magnetic field with the help of permanent magnets fixed on a rotating plate while applying differentiation markers to the macrophages. The results were obtained using microscopy, flow cytometry, and ELISA. In the flow cytometry and ELISA figures, PAM and MAD represent the PAM surface and _Mad_Surface, respectively, consistently across all figures in the paper. (B) SEM images of a PAM surface (left) and _Mad_Surface (right). (C) The measured pulsed magnetic field of ∼50 mT at an applied frequency of 0.1 Hz at the surface of hydrogel samples placed on the setup depicted in panel A. (D) Surface stiffness changes on PAM and _Mad_Surface under static and dynamic conditions. (E) Viability of M0 macrophages on PAM and _Mad_Surface under static and dynamic conditions, based on live–dead staining.

We cultured macrophages on hydrogel surfaces fabricated in 3-well Ibidi chips. Figure 1A is the schematic overview of the experimental setup with a pulsed magnetic field and the positioning of the chips and hydrogel surface, e.g., _Mad_Surface consists of magnetic microbead-embedded polyacrylamide hydrogels (Figure 1B). A rotating plate equipped with four sets of magnets was placed underneath the fixed stage to control the frequency of the magnetic field applied to the _Mad_Surface and cells. The size of the magnets was chosen to match the size of the wells in Ibidi chips. The pulsed magnetic field measurement using a Gauss meter (Figure 1C) indicated an increase in magnetic flux density from 0 to ∼50 mT at the hydrogel surface upon exposure to intermittent durations of 10 s. The chosen time interval was selected to synchronize with the peak duration of the magnetic field as it reaches the center of the hydrogel surfaces (Figure 1C).

We characterized the hydrogel surfaces using scanning electron microscopy (SEM) to obtain insight into the structural morphology of the hydrogel surface (Figure 1B). PAM samples displayed a relatively uniform granular appearance, indicating successful cross-linking of the material (Figure 1B, left). The surface was generally uniform, where the presence of the nano and micropores contributed to the overall texture and potential functionalities of the hydrogel in terms of interaction with biological entities.^25^ To assess the effect of an external magnetic field on macrophage phenotype, we used micrometer-sized magnetic beads which reduce the chance of engulfment into the macrophages. SEM analysis (Figure 1B, right panel) showed a well-defined, nonactuated _Mad_Surface, where magnetic microparticles are uniformly dispersed and well-embedded throughout the hydrogel matrix, providing consistent coverage over the particles.

Macrophages experience dynamic changes in matrix stiffness under various physiological conditions, varying from 10^–1^ kPa range in the human brain to >100 kPa range in dermal tissue and bone, which significantly impacts their function and phenotype.^26,27^ The stiffnesses of PAM and the _Mad_Surface were evaluated (Figure 1D), where we did not observe changes in the stiffness of PAM surfaces under dynamic conditions. Young’s modulus was measured as 100 kPa, which is considered a stiff surface for biological cells.^28,29^ Yan *et al.* and Blakney *et al.* have reported that macrophages can distinguish stiffness change in the range of 100 to 300 kPa and appear more spread on stiffer substrates.^30,31^ We measured the stiffness of _Mad_Surface as 172 kPa under the condition without MF, which is found to be significantly higher than the PAM surface in the same conditions (Figure 1D). This increased stiffness can be attributed to the presence of embedded magnetic microparticles within the hydrogel matrix. In the condition of with MF, the stiffness of the _Mad_Surface increased 1.9 times, from 172 to 336 kPa in the bulk. The increase in stiffness under MF conditions is a direct result of the mechanical properties of the magnetic microparticles, the alterations in their orientation, and interactions when influenced by an external magnetic field.^29,30^ An increase in a similar stiffness range has been shown to increase the metabolic activities of fibroblasts and stem cells while each different tissue has its own dynamic stiffness characteristics.^28,32^ Although it is challenging to find the technology needed to experimentally measure the dynamic change in hydrogel stiffness as we initially hypothesized, modeling work has reported that the stiffness of a 200 μm thick hydrogel rapidly responds to a 300 kA/m magnetic field, which is approximately 3.77 mT within 4 s and reaches an equilibrium state within 10–14 s.^33,34^ Given the fact that we applied a magnetic field of ∼50 mT to 100 μm thick hydrogel surfaces, we assume that within a 10 s interval, the stiffness of the hydrogel in our experiment will reach an equivalent state when placed in a pulsatile magnetic field or in a constant magnetic field with the same flux density.

Next, we characterized the surface stiffness of PAM and _Mad_Surface. The characterization took place with and without the magnetic field actuation, termed in this study as magnetic + and magnetic – conditions, respectively. The magnetic + condition was applied in pulsatile format and not continuously to enhance the dynamicity of the platform. The effect of constant magnetic field application was previously studied on immune cells.^35^ For magnetic + conditions, hydrogel surfaces were placed under the magnetic field with a magnetic flux density of ∼50 mT. Magnetic fields have been classified based on their intensity into four categories: weak (<1 mT), moderate (1 mT to 1 T), strong (1–5 T), and ultrastrong (>5 T) for biological organisms.^36^ In this scale, 50 mT falls into the lower end of the moderate strength and it has been shown to relatively increase proliferation and differentiation in different cell types.^37^ Strong and ultrastrong intensities have been shown to link with adverse effects on cell viability.^38,39^ We applied the 50 mT static magnetic field with 0.1 Hz frequency (Figure 1C) (which is considered a low frequency) previously shown to promote cell adhesion to the substrates. Similar magnetic field frequency has been shown to stimulate the adhesion and M2-polarization of macrophages.^40^ At this magnetic field condition, particles align themselves with the magnetic field direction by deforming the hydrogel locally, leading to topographical changes.^30^ Accordingly, we hypothesized that the cells would adhere to the substrate, while they can differentiate thanks to the combined effect of the magnetic field and stiffness changes.

To assess cell viability and adherence on hydrogel surfaces, we conducted cell culturing experiments involving PAM and _Mad_Surface with and without pulsed magnetic field, termed in this study as dynamic and static conditions, respectively, where PAM and static conditions served as a control group. As initial cell attachment was not observed on the unmodified PAM hydrogel surfaces, these surfaces were precoated with collagen type 1 a day before cell seeding.^16^ Following cell seeding, a settling period of 24 h was allowed before the application of the static and dynamic conditions. Figure 1E shows the macrophage viability on these surfaces. Macrophages that attached to the surfaces were collected and evaluated using flow cytometry based on biomarker staining. Across all tested conditions, the cell viability was consistently not below 85%, indicating the absence of cytotoxic signals of these substrates that compromise cell survival. To validate the methodology, a 1:1 mixture of live and dead cells served as a positive control for cell death. The differences in cell viability on PAM and _Mad_Surface under both static and dynamic conditions were not found to be statistically significantly different.

We evaluated the attachment of cells to the surfaces through fluorescence microscopy, staining M0 naïve THP-1 macrophages with phalloidin for the cytoskeleton and DAPI for the nucleus. Figures S1A and S1C demonstrate that cells attached more on the PAM surface when compared to the _Mad_Surface under static conditions. After 24 h of pulsed magnetic field application, a reduction in cell attachment on PAM and _Mad_Surface was observed compared to that under the static condition (Figure S1B and S1D). Phalloidin staining reveals stress fiber formation at the outer boundaries of the cytoskeleton in all conditions, which might be attributed to the cells’ reduced response to topographical changes in comparison to their magnetic field response. The reduced amount of cells on _Mad_Surface compared to PAM could be attributed to the surface microroughness comparable to the cell size and the effect of the magnetic field.^41,42^ Compared to the smooth PAM surface, more microroughness was observed on the _Mad_Surface due to the existence of magnetic microbeads (Figure 1B).

### 2.2 Bulk Population Analysis of Polarized Macrophages

Intrapopulation heterogeneity denotes variances among subgroups of macrophages derived from the same source and it is given rise by differentiation and modulation.^43^ To minimize batch-to-batch variability in primary macrophages and isolate the effects of the magnetic field and stiffness changes, we utilized THP-1 macrophages, known for their demonstrated heterogeneity in previous studies.^44,45^ We sought to explore the macrophage response to PAM and _Mad_Surface induced by magnetic field application under static and dynamic conditions. Our analysis for the characterization of the bulk population is based on flow cytometry and ELISA.

**Figure 2:**
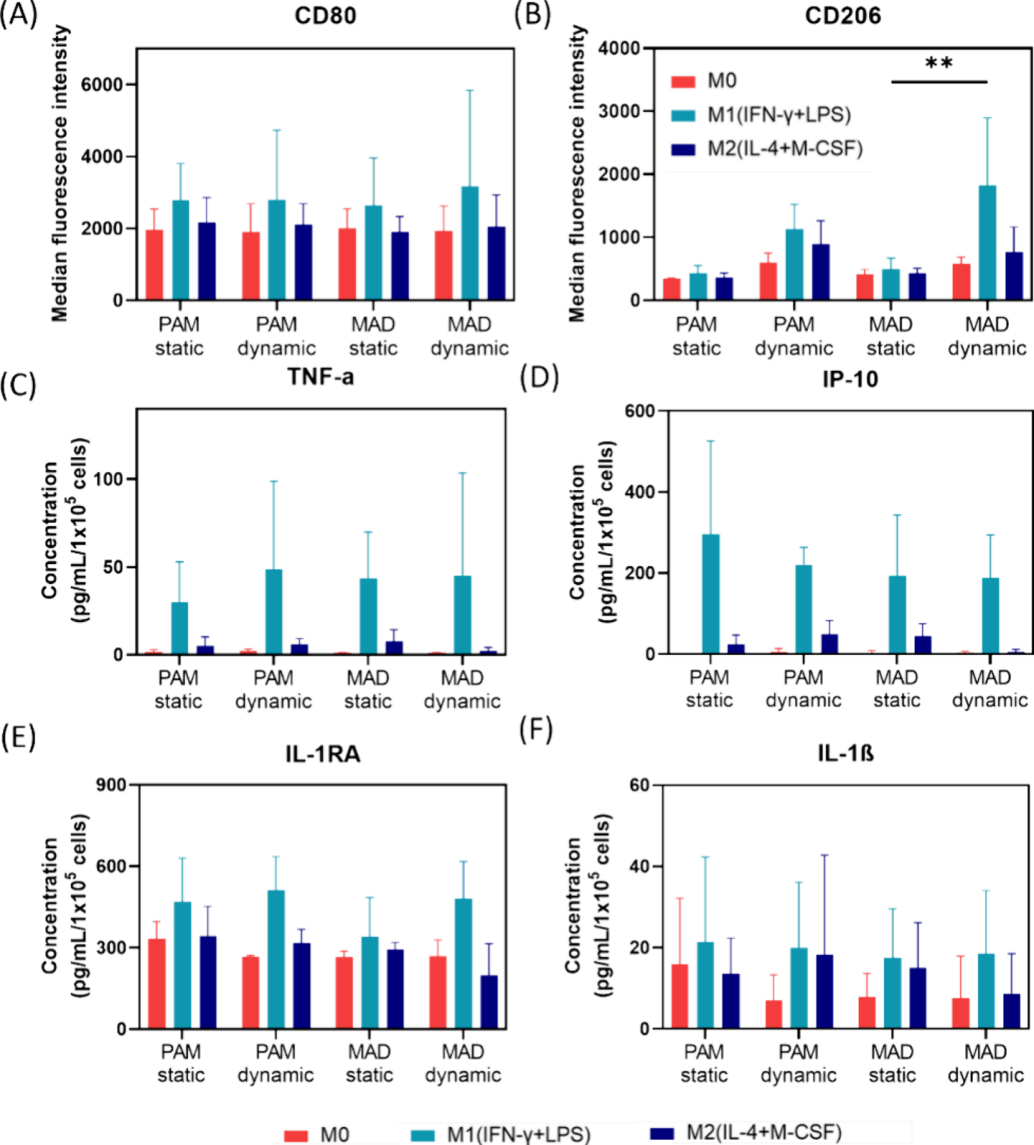
Characterization of polarized macrophages in bulk. (A and B) Flow cytometry analysis and (C–F) ELISA results for TNF-α, IP-10, IL-1RA, and IL-1β cytokines. (A) Flow cytometry data (shown in MFI) of CD80 and (B) CD206 stained macrophages collected from PAM and _Mad_Surface under static and dynamic conditions. Values shown are mean values ± SD (*n* = 3). ELISA results were obtained by using cell supernatants from polarized macrophages on PAM and _Mad_Surface under static and dynamic conditions. The concentrations of (C) TNF-α, (D) IP-10, (E) IL-1RA, and (F) IL-1β cytokines secreted by M0-, M1-, and M2-activated macrophages on PAM and _Mad_Surface under static and dynamic conditions. Values shown are mean values ± SD (*n* = 4), **refers to *P* < 0.001.

Using flow cytometry, we measured CD80 and CD206 antibody expressions as a marker for M1 and M2 macrophage phenotypes, respectively. Figure S3 displays representative histograms of surface markers CD80 and CD206 expressed by M0-activated, M1-activated (M0 macrophages activated by IFN-γ and LPS), and M2-activated (M0 macrophages activated by IL-4 and M-CSF) macrophages cultured on PAM and _Mad_Surface under static and dynamic conditions, as analyzed by flow cytometry. Median fluorescent intensity (MFI) measurements indicate the average fluorescence intensity of the marker associated with a particular macrophage phenotype.^46^Figure 2A shows the MFI of CD80 expressed by cells that were cultured on PAM and _Mad_Surface under static and dynamic conditions. On the PAM surface under static conditions, CD80 expression in M0-activated, M1-activated, and M2-activated macrophages showed no statistically significant difference in MFI compared to M0-activated, M1-activated, and M2-activated macrophages cultured under dynamic conditions. Comparable CD80 levels under static conditions were observed on _Mad_Surface, with MFI values of 2007, 2642, and 1903 au expressed by M0-activated, M1-activated, and M2-activated macrophages, respectively. Thus, no statistically significant differences were observed between these conditions.

Figure 2B shows the MFI of the M2 marker CD206. On both PAM and _Mad_Surface surfaces, M0-activated, M1-activated, and M2-activated macrophages showed comparable CD206 expression levels under static conditions. Upon exposure to the pulsed magnetic field, the expression of the M2 marker CD206 increased on both surfaces. We also observed this trend when comparatively analyzing the M1 over M2 marker ratio for the given conditions (Figure S4A). On the PAM surface, an increasing trend in CD206 expression level was observed in all types of stimulated macrophages. Notably, the expression level of CD206 was significantly increased in M1-activated macrophages when cultured on the _Mad_Surface under dynamic conditions (M0-activated = 586 au, M1-activated = 1823 au, M2-activated = 765 au), compared to M1-activated macrophages cultured on the same surface in static conditions. Here, the increased expression of CD206 in M1-activated macrophages on _Mad_Surface can indicate a phenotypic shift toward a more M2-like phenotype under the influence of the pulsed magnetic field. Our finding is supported by previous research showing that the magnetic field application reversibly stimulated the NADH–oxidase pathway in macrophages and their precursors via the increased superoxide anion radicals.^47,48^ In another work, the magnetic field has been shown to induce the M2 phenotype over M1,^49,50^ which also supports our observations. To further investigate the impact of the dynamic behavior exhibited by the applied magnetic field on the _Mad_Surface, we conducted the same experiment under a constant magnetic field of 50 mT, termed as the “constant” condition in Figures S5A and S5B. Results show a higher CD80 expression level of 2847, 5001, and 2891 au by M0-, M1-, and M2-activated macrophages cultured on _Mad_Surface in constant magnetic field compared to static (with MFI values of 2007, 2642, and 1903 au for M0-, M1-, and M2-activated macrophages, respectively) and dynamic conditions (with MFI values of 1943, 3171, and 2038 for M0-, M1-, and M2-activated macrophages, respectively) (Figure S5A). This could suggest that a constant magnetic field may trigger a shift toward an M1 phenotype, differing from the outcomes observed under pulsed magnetic field conditions. The expression level of CD206 in macrophages on _Mad_Surface showed no significant variation when comparing constant conditions to either dynamic or static conditions; however, a decrease in CD206 expression level from 1823 to 1339 au was noted when comparing constant to dynamic conditions, indicating that the _Mad_Surface in the dynamic condition might result in a more pronounced shift toward the M2 phenotype within the bulk population than the constant condition.

We performed multiplex ELISA to evaluate the polarization states of the bulk macrophage cell population, aiming to validate the findings obtained from flow cytometry. We opted for this approach to directly observe the impact of dynamic conditions on cells through protein secretion. In our experimental setup, THP-1 macrophages were cultured again in the presence of M0, M1, and M2 stimuli on both PAM and _Mad_Surface under both static and dynamic conditions. By analyzing cell culture supernatant, we assessed the secretion levels of 10 different cytokines, of which only TNF-α, IP-10, IL-1RA, and IL-1β were secreted at detectable levels (Figures 2C–2F). From these cytokines, IL-1RA is categorized as an anti-inflammatory (M2) cytokine, whereas TNF-α, IP-10, and IL-1β belong to the category of pro-inflammatory (M1) cytokines. Although a trend of increased cytokine secretion was observed in the M1-activated macrophages compared to the M0-activated macrophages, we did not observe any statistically significant differences in cytokine levels between the static and dynamic conditions for the PAM or _Mad_Surface. We also confirmed this by comparatively analyzing the M1:M2 ratio in Figure S4B. In accordance with the flow cytometry analysis, no significant differences in the polarization state could be observed at the bulk population level. It is likely that the increase in stiffness of the _Mad_Surface under dynamic conditions is not sufficient to induce an observable change in macrophage phenotype, which has also been described in previous studies comparing surfaces in a similar stiffness range.^31,51^

### 2.3 Single-Cell Analysis of Polarized Macrophages

Macrophages, known for their adaptable nature, demonstrate heterogeneous phenotypes and functions typically classified on an M1 to M2 spectrum.^43^ Macrophages can exhibit a mixed or transitional phenotype, expressing markers associated with both M1 and M2 states.^52^ To gain further insights into the expression of macrophage markers associated with different macrophage phenotypes, we investigated the percentage of positive cells (PPC) providing information about the proportion of cells expressing a specific marker by gating the flow cytometry data of Figure 2. We defined the cell polarization status by the percentage of CD80- and CD206-positive cells (Figure S3).

**Figure 3:**
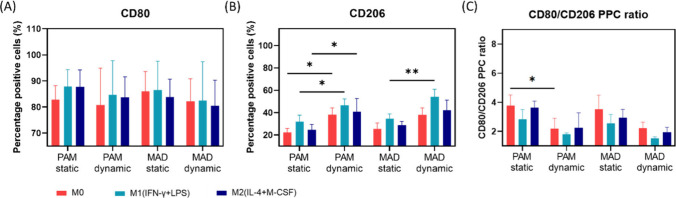
Characterization of polarized macrophages at the single-cell level. Flow cytometry analysis of (A) the percentage of CD80-positive cells, (B) the percentage of CD206-positive cells, and (C) the CD80/CD206 PPC ratio of macrophages collected from PAM and _Mad_Surface under static and dynamic conditions. Values shown are mean values ± SD (*n* = 3). *Refers to *P* < 0.05 and **refers to *P* < 0.001.

Figures 3A and 3B show differences in the polarization of macrophages on the PAM surfaces and _Mad_Surfaces. Under static conditions on PAM surfaces, 22% of the M0-activated cells, 32% of M1-activated cells, and 25% of M2-activated macrophages were CD206-positive. Similarly, on the _Mad_Surface, the percentages are 25% for M0-activated, 34% for M1-activated, and 29% for M2-activated cells. Here, the initial stiffness difference between the two surfaces did not induce statistically significant differences in M2 marker expression, indicating no shift in macrophage polarization states. However, upon exposure to a pulsed magnetic field, M2 marker CD206 expression increased significantly on the PAM surface, with percentages of M0-activated, M1-activated, and M2-activated macrophages expressing CD206 at 38%, 47%, and 41%, respectively. This indicates a notable shift efficiency at which exposed cells are changing their phenotypes toward M2 when the pulsed magnetic field is applied for 24 h. This duration has previously been found sufficient for initiating the modulation of individual macrophage phenotypes but not yet at the population level.^53,54^

On the _Mad_Surface, the percentage of CD206-positive cells was measured as 38%, 54%, and 42% for M0-activated, M1-activated, and M2-activated macrophages, respectively, under dynamic conditions. For the M1-activated macrophages, this percentage was significantly higher than under static conditions. In the M0- and M2-activated macrophages, we also observed an increasing trend, but this was not statistically significant. Conversely, the expression of the M1 marker CD80 showed no significant difference in the percentage of positive cells among M0-activated, M1-activated, and M2-activated macrophages cultured on both PAM and _Mad_Surface (Figure 3A).

The PPC ratio of CD80- and CD206-positive cells in Figure 3C shows a general bias toward the M2 phenotype among the M0-, M1-, and M2-activated macrophages under dynamic conditions on both the PAM and _Mad_Surface, although this was only significant for M0-activated macrophages on the PAM surface. These results suggest that the pulsed magnetic field regulates macrophage polarization toward the M2 phenotype. Additionally, under dynamic conditions, the _Mad_Surface intensified this phenotypic shift in both M1-activated and M2-activated macrophages compared with the PAM surface, as evidenced by the decrease in the PPC ratio from 1.8 to 1.5 for M1-activated macrophages and from 2.2 to 1.9 for M2-activated macrophages. Again, we conducted the same experiments in constant conditions where M0-, M1-, and M2-activated macrophages were cultured under a constant environment, with a continuous application of a 50 mT magnetic field throughout the experiment, and results are shown in Figures S5C and S5D. It was observed that the expression level of CD80 increased among the M0-, M1-, and M2-activated macrophages maintained in a constant environment, showing PPC values of 94.8%, 96.2%, and 96.9% for M0-, M1-, and M2-activated macrophages, respectively. This was in contrast to the macrophages in dynamic conditions, which displayed lower PPC values of 82.2%, 82.5%, and 80.4% for M0-, M1-, and M2-activated macrophages, respectively. On the other hand, there was a minor reduction in the expression level of CD206 for M0- and M2-activated macrophages in constant condition (with PPC values of 31.3% and 33.7% for M0- and M2-activated macrophages, respectively) compared to dynamic conditions (with PPC values of 37.9% and 42.1% for M0- and M2-activated macrophages, respectively). Meanwhile, the expression level of CD206 in M1-activated macrophages remained relatively stable in both constant (with a PPC value of 54.0%) and dynamic conditions (with a PPC value of 51.5%). The results are in line with the study on MFI in Figures S5A and S5B, where the _Mad_Surface placed in a constant magnetic field results in a phenotypic shift of macrophages toward the more pro-inflammatory phenotype compared to the condition in which _Mad_Surface is placed in a pulsed magnetic field in our experiment.

Overall, we found that 24 h application of a 50 mT magnetic field with a 0.1 Hz frequency modulates macrophages toward the M2 phenotype. This was observed in flow cytometry, but not in ELISA results. This effect could be best observed in a subpopulation of the activation conditions at the single-cell level, wherein particular M1-activated macrophages showed a stronger bias toward the M2 phenotype (increased percentage of CD206-positive cells) under dynamic conditions on both surfaces when compared to M0. An additional effect for the change in stiffness of the _Mad_Surface under dynamic conditions pronounced the shift toward the M2 phenotype, as a decrease in CD80/CD206 PPC value was observed compared with those observed on the PAM surface under dynamic conditions. Noticeably, we observed that the application of a pulsed magnetic field exerted a more pronounced influence on macrophage phenotype compared to alterations in substrate stiffness within the range examined and with a constant magnetic field. The _Mad_Surface approach has the potential to establish controlled environments, enhancing our understanding of the interactions among surface mechanics, magnetic field application, and macrophage behavior.

## 3 Conclusions

Macrophages are key regulators in tissue regeneration as they undergo a shift in cytokine secretion profile from an M1 pro-inflammatory in the initial stages of wound healing to an M2 anti-inflammatory gradually at the later stages. This study explores the use of magnetically actuated dynamic polyacrylamide hydrogel surfaces as a hybrid approach to dynamically and reversibly modulate the cytokine secretion profiles in macrophages within the *in vitro* cell culture microenvironment. We used _Mad_Surfaces with dynamic and reversible stiffness alterations in response to a pulsed magnetic field with a frequency of 0.1 Hz and a magnetic field flux density of ∼50 mT. We observed that M1 pro-inflammatory macrophages demonstrated an increased expression of CD206, resulting from a higher percentage of CD206-positive cells upon magnetic stimulation. Exploring the design space of the experimental model further can improve the effect size of the macrophage response. In this way, the current limitations in this work (e.g., a broader range of stiffnesses, pulse frequencies, magnetic field flux densities, and exposure times, as well as focal adhesion markers of macrophages) can be addressed. The ability to efficiently and noninvasively alter macrophage cytokine secretion profiles using the _Mad_Surface approach presents a step toward controlling immune reactions in tissue regeneration.

## 4 Materials and Methods

### 4.1 Fabrication of _Mad_Surface

The _Mad_Surface was fabricated in 3-well glass bottom Ibidi chips (cat. no.:8038, Ibidi GmbH, Gräfelfing, Germany); a schematic drawing of the fabrication process is shown in Figure S2. First, the glass surface of the Ibidi chip was methacrylated using 3-(trimethoxysilyl)propyl methacrylate (Merck Life Science NV, Amsterdam, The Netherlands) via the protocol reported in our previous work.^55^ A polyacrylamide hydrogel solution was prepared by mixing 40 wt % acrylamide solution and 1.8 wt % bis-acrylamide monomers with a 3 mg/mL magnetic microparticle (Carboxyl Ferromagnetic Particles, monodisperse, 4–4.9 μm in diameter) solution at a volume ratio of 1:1:1. The solution was mixed thoroughly using a magnetic stirrer to ensure homogeneity. To initiate the polymerization process, a photoinitiator, 2-hydroxy-2-methylpropiophenone (Sigma-Aldrich, The Netherlands) was added to the hydrogel solution at a volume ratio of 1:100 to achieve efficient cross-linking. The prepared hydrogel solution was carefully pipetted into the wells of the Ibidi 3-well glass bottom chip. A 13 mm round-shaped glass coverslip was placed on top of the hydrogel precursor droplet to obtain a thickness of 100 μm consistently. The hydrogel solution in the chip was exposed to ultraviolet (UV) light using a UV-LED exposure system (UV-EXP 150s, IDONUS, Switzerland) at a light intensity of 40 mJ/cm^2^ and a dose of 500 mJ/cm^2^. Afterward, the coverslip was removed carefully.

### 4.2 Structural Characterization of _Mad_Surface

The surface profile and stiffness of PAM surfaces and _Mad_Surfaces were characterized by scanning electron microscopy and a Nanoindenter (Piuma Optics11 Life), respectively. For the scanning electron microscope (SEM) characterization, hydrogel samples were cut into small sections, fixed onto SEM stubs, and freeze-dried (LABCONCO, FreeZone 4.5) under a pressure of 0.02 mbar and at −54 °C overnight. To prevent charging during imaging, the dried samples were coated with a thin layer of gold using a sputter coater (Quorum Q300T D Plus). The porous structures of freeze-dried hydrogels were imaged using SEM (FEI ESEM Quanta 600) at an acceleration voltage of 5 kV. The stiffness characterization of the polyacrylamide hydrogel was conducted using the nanoindenter. Initially, hydrogel samples fabricated on glass slides were placed in PBS buffer solution in a glass Petri dish. A cantilever with a stiffness of 3.5 N/m and a tip radius of 25 μm was used to measure the stiffness of the hydrogel samples. To measure the stiffness of the hydrogel surface under the magnetic field, a permanent magnet was placed underneath the Petri dish, where the distance between the magnet and the Petri dish was adjusted to achieve a magnetic flux density of ∼50 mT at the surface of the hydrogel sample in air.

### 4.3 Measurement of Magnetic Flux Density at Hydrogel Surfaces

The magnets (Q-15-15-03-N, Supermegnete, The Netherlands) used in our study have dimensions of 2.0 × 2.0 cm, slightly larger than the Ibidi chip wells, which measure 1.65 × 1.65 cm, and significantly larger than the hydrogel surface with a diameter of approximately 1.0 mm. These magnets were securely fixed in a central alignment with the wells and hydrogel samples on an electronic rotating plate (p2717585, Cameranu.nl) that rotated at a constant speed of 40 s/cycle to ensure consistent exposure to the magnetic field. To maintain uniformity in the magnetic field strength across all three wells of the Ibidi chip, the magnets were spaced apart at a specific distance. The magnetic field intensity was measured at the center of the hydrogel using a Tektronix oscilloscope (TDS 210, two-channel digital real-time oscilloscope) in conjunction with a Gaussmeter (FW Bell Model 811a Digital Gaussmeter) to achieve a flux of 50 mT at a frequency of 0.1 Hz.

### 4.4 Collagen Coating and Sterilization of Hydrogel Surfaces

After polymerization, the chip was carefully rinsed with deionized water to remove any unreacted monomers, photoinitiator residue, or impurities. Sterilization was then performed using standard UV sterilization in a biosafety cabinet for 20 min to ensure aseptic conditions for subsequent cell culture experiments. The hydrogel surface was then functionalized with Sulfo-Sanpah followed by cell-surface protein, collagen type I, cross-linking at a concentration of 100 μg/mL at 4 °C overnight. A thorough washing step using sterilized deionized water was performed after coating to remove any unreacted collagen molecules. Before the freshly coated hydrogel surfaces were used for cell seeding, another UV sterilization of 15 min was performed right after the washing step to avoid contamination. The experimental procedure was uniform across all samples, resulting in a constant impact on the level of the pathogenic residue. The collagen coating is not expected to affect the stiffness of the surface felt by the cells. The previous work has demonstrated that collagen type I bound to gel surface does not impact the substrate stiffness.^56,57^

### 4.5 Differentiation of THP-1 Monocytes into Macrophages and Polarization on Hydrogel Surfaces

THP-1 cells (ATCC-TIB-202, LGC Standards GmbH) were cultured in RPMI-1640 medium (Merck Life Science NV) supplemented with 10% fetal bovine serum (Merck Life Science NV) and 1% penicillin–streptomycin (Thermo Fisher Scientific) at a density of 400,000 cells/mL. To induce macrophage differentiation, THP-1 cells were treated with 50 ng/mL phorbol 12-myristate 13-acetate (PMA, Merck Life Science NV) for 24 h in a T75 culture flask. Following PMA treatment, the differentiated THP-1 cells were detached from the T75 flask surface using 10 mL Accutase (Merck Life Science NV), harvested, and seeded onto _Mad_Surface, which is attached to the glass bottom of the 3-well Ibidi chip, at a seeding density of 190,000 cells/cm^2^ in a final volume of 1 mL of culture medium per well. In order to get enough cells for further analysis, cells were seeded on three surfaces located in three wells with the same culture conditions applied. The macrophages were allowed to settle on the _Mad_Surface for 24 h. To promote the M1 pro-inflammatory phenotype, THP-1 macrophages on _Mad_Surface were exposed to 10 ng/mL lipopolysaccharide (LPS, Merck Life Science NV) and 10 ng/mL recombinant human interferon-γ (IFN-γ, Peprotech EC, Ltd.). Conversely, to induce an M2 anti-inflammatory phenotype, 20 ng/mL interleukin-4 (IL-4) and 50 ng/mL recombinant human M-CSF (Peprotech EC, Ltd.) were added to the culture media for 24 h.^58^ During the polarization process, the _Mad_Surface was magnetically stimulated at a pulsatile frequency of 0.1 Hz to modulate the stiffness of the surface. The dynamic manipulation allowed for precise control of the mechanical properties experienced by the macrophages. As the negative controls, cells were cultured on PAM surfaces under the same dynamic culture conditions and under static conditions separately.

### 4.6 DAPI/Phalloidin Staining

Cells were fixed with a 4% paraformaldehyde (Fisher Scientific) solution for 15 min. Cell membranes were permeabilized in 0.1% Triton X-100 (Merck Life Science NV). The cytoskeleton was stained using a 1:40 dilution of Alexa Fluor 488 conjugated phalloidin (Merck Life Science NV) in PBS, and nuclei were stained using a 1:1000 dilution of DAPI (Merck Life Science NV). Cells were imaged at three to five randomly selected regions using Leica DM-i8 at 20× magnification.

### 4.7 Cell Marker Staining and Flow Cytometry Analysis

Following the designated polarization period, the THP-1 macrophages were detached from the _Mad_Surface by incubating in 1 mL/well Accutase in the incubator for 1 h; cells that were cultured under the same condition in different wells were pooled together after detachment. After collecting the cells, cells were isolated and suspended to a concentration of 100,000 cells/mL in a final volume of 300 μL of phosphate-buffered saline (PBS). Following cell isolation and suspension preparation, separate tubes were designated for FITC antihuman CD80 antibody and APC/Cyanine7 antihuman CD206 (MMR) antibody (BioLegend Europe B.V.) staining with an incubation time of 30 min, followed by a thorough washing to remove unbound antibodies, after which the cells were optionally fixed. Subsequently, flow cytometry analysis was conducted using a BD FACSCanto II instrument, where compensation controls and negative controls were set using single-stained samples including CD80 single-stained, CD206 single-stained, and unstained controls to adjust spectral overlap and determine the threshold for positive events, respectively. Results are shown in Figures S3I and S3J. Gating based on forward and side scatter excluded debris, allowing for the evaluation of CD80 and CD206 expressions within the macrophage population as well as single macrophages.

### 4.8 Cell Viability Test

To assess the cell viability, the Zombie Red Fixable Viability Kit (BioLegend) was utilized. Following the completion of experimental treatments, cells were harvested and resuspended in PBS at a concentration of 300,000 cells/mL. Subsequently, the Zombie Red dye was added to the cell suspension at a final dilution of 1:300, and cells were incubated for 20 min at room temperature in the dark, followed by washing steps and subsequent flow cytometry analysis. As a positive control, a mixture containing a 1:1 ratio of live cells and cells treated with 70% ethanol for 15 min was used.

### 4.9 Cytokine Secretion Measurement and Quantification

Cells were cultured in three wells on either PAM surfaces or _Mad_Surfaces for each specific culture condition. After culturing and polarization, 300 μL of culture medium (100 μL/well) was pooled for each condition and centrifuged at 3000 rcf for 10 min. Subsequent to centrifugation, 150 μL of supernatant was collected and stored at −80 °C for subsequent cytokine analysis.

Quantification of secreted cytokines was conducted using the 13-plex LEGENDplex Human Macrophage/Microglia panel (BioLegend) designed for the identification of IL-12p70, TNF-α, IL-6, IL-4, IL-10, IL-1β, Arginase, CCL17 (TARC), IL-1RA, IL-12p40, IL-23, IFN-γ, and CXCL10 (IP-10). Of these, arginase, IFN- γ, and IL-4 were excluded from analysis due to the high background levels in the culture medium. The assay was carried out according to the manufacturer’s instructions, with the following adjustments. Five microliters of the sample or standard was mixed with 5 μL of assay buffer, beads, and detection antibodies in V-bottom polypropylene 96-well plates and incubated at room temperature (shaking at 800 rpm). Following a 2 h incubation period, 5 μL of streptavidin–phycoerythrin (SA-PE) dye was added to each well, and the plates were incubated for an additional 30 min. Subsequently, wells were washed with 150 μL of wash buffer, followed by centrifugation at 1000 × g and removal of the supernatant. After the beads were resuspended in 80 μL of wash buffer, fluorescence intensity was assessed using a BD FACSCanto II flow cytometer. The acquired data was analyzed using the Biolegend LEGENDplex Data Analysis Software Suite.

### 4.10 Statistical Analysis

Flow cytometry and ELISA data were analyzed through a two-way analysis of variance (ANOVA) to assess the influence of two independent variables: biochemical stimulation (M0, M1 (IFN-γ + LPS), or M2 (IL-4 + M-CSF) and surface types (PAM-static, PAM-dynamic, MAD-static, and MAD-dynamic) on the observed outcomes. The data set was derived from three independent experiments. The ELISA data were normalized to cell count (Table S6) prior to analysis. Stiffness and viability data, characterized by a singular independent variable (surface type), were analyzed via a one-way ANOVA. Posthoc comparisons were facilitated by Tukey’s honest significant difference (HSD) test in instances where significant differences were identified. The predetermined significance levels were denoted as *p < 0.05 and **p < 0.001. All statistical analyses were executed using GraphPad Prism 9. For calculation of the M1/M2 cytokine ratio, individual cytokine data sets were first standardized by dividing through the standard deviation before calculating the ratio (IL-1β + TNF-α + IP-10)/(IL-1RA).

## Supporting Information Available

Additional experimental details, materials, and methods, including photographs of experimental setup; M0 macrophage cell attachment and viability on hydrogel surfaces; representative flow cytometry histograms of surface biomarkers; cell number collected from hydrogel surfaces after detachment; and characterization of polarized macrophages in bulk (PDF)
