# Supplementary material for: Magnetically Driven Hydrogel Surfaces for Modulating Macrophage Behavior
Source: ACS Biomater Sci Eng. 2024 Oct 9;10(11):6974–83. doi: 10.1021/acsbiomaterials.4c01624 (PMC11558558; doi:10.1021/acsbiomaterials.4c01624)
Supplement: Supplementary file 1 — ab4c01624_si_001.pdf [file ab4c01624_si_001.pdf]

Supporting Information

Magnetically-Driven Hydrogel Surfaces for Modulating Macrophage Behavior

Lanhui Li[1,2], Els Alsema[2,3], Nick R.M. Beijer[3], and Burcu Gumuscu[1]*

[1] Biosensors and Devices Lab, Institute for Complex Molecular Systems, Eindhoven University of Technology, 5600 MB, Eindhoven, the Netherlands

[2] Biointerface Science Group, Institute for Complex Molecular Systems, Eindhoven University of Technology, Eindhoven, 5600MB, Eindhoven, the Netherlands

[3] Centre for Health Protection, National Institute for Public Health and the Environment (RIVM), 3720BA, Bilthoven, the Netherlands

* Corresponding Author: b.gumuscu@tue.nl; Tel: +31 (0)40 2478373

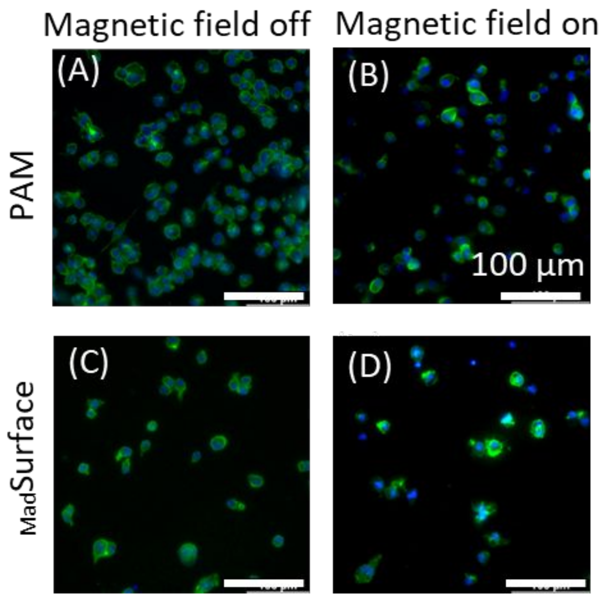

Figure S1. M0 macrophage cell attachment and viability on hydrogel surfaces. DAPI/Phalloidin staining of M0 macrophage on PAM in the condition (A) without magnetic field (static) and (B) with pulsed magnetic field (dynamic) and $_{\mathrm{Mad}}$Surface under (C) static and (D) dynamic conditions.

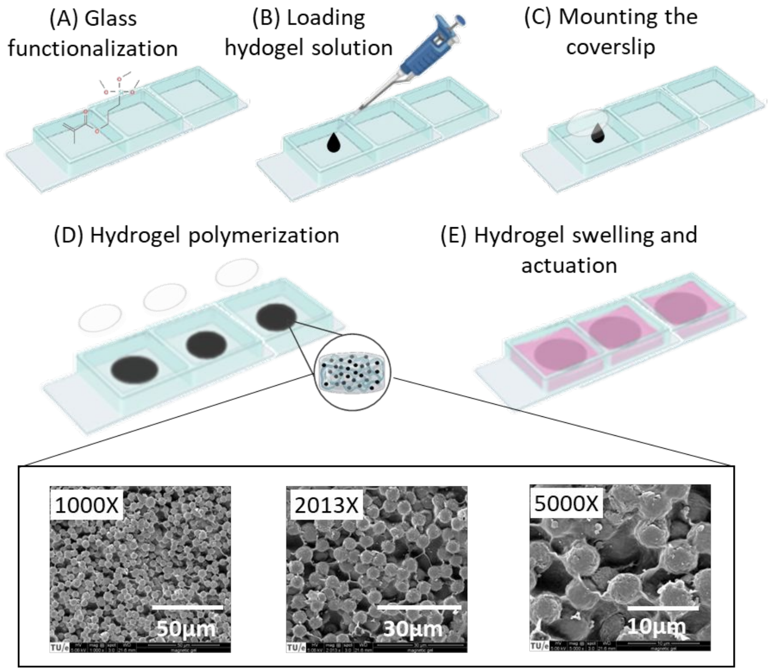

Figure S2. Schematic drawing of the fabrication of $_{\mathrm{Mad}}$Surface in 3 well glass bottom Ibidi chips and SEM images of $_{\mathrm{Mad}}$Surface at varying magnifications.

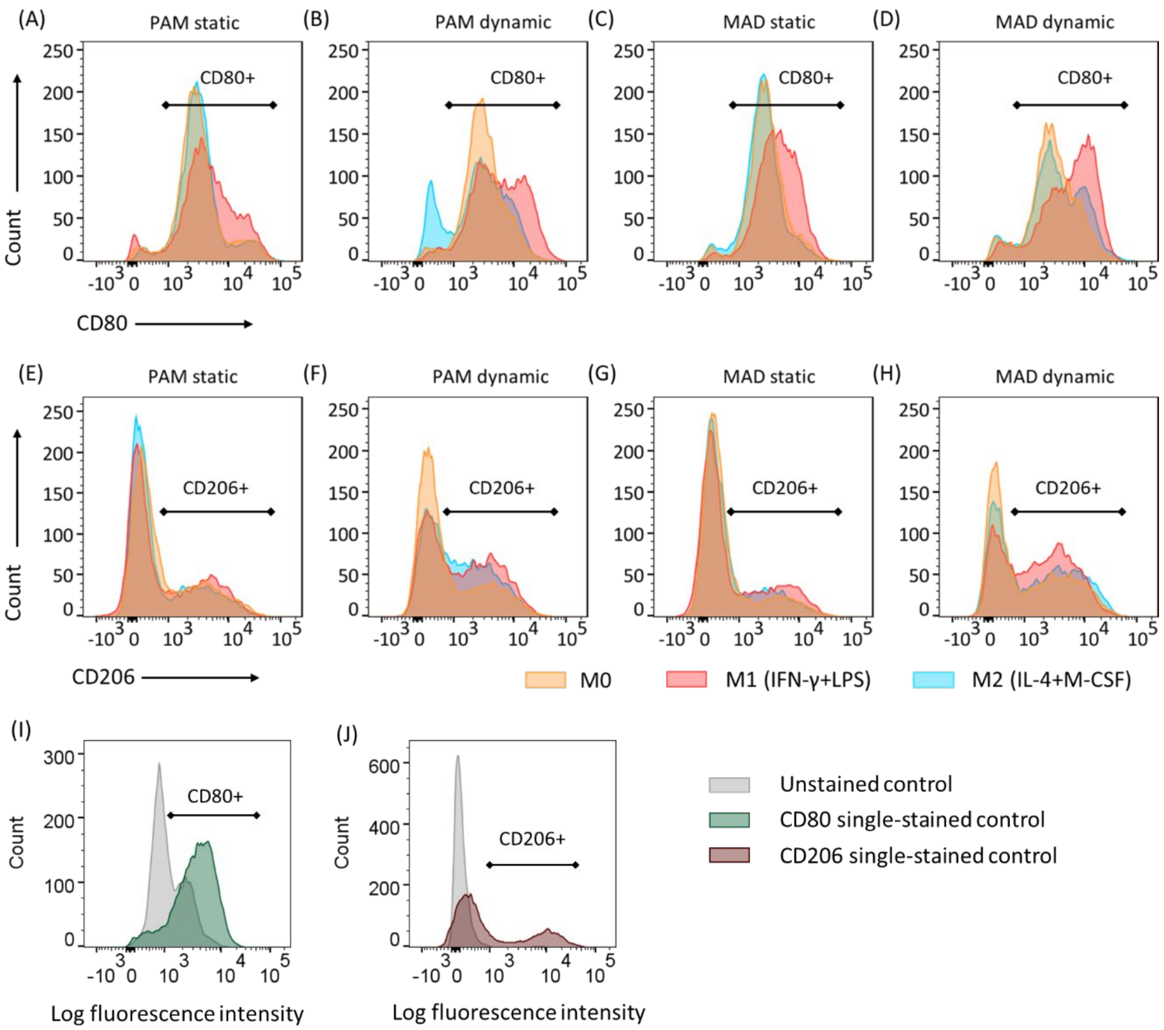

Figure S3. Representative histograms of surface marker CD80 (M1 macrophage marker) (A-D) and CD206 (M2 macrophage marker) (E-H) of M0-activated, M1-activated and M2-activated macrophages on PAM and $_{\text{Mad}}$Surface in static and dynamic conditions analyzed by flow cytometry. (I) and (J) represent histograms of unstained, CD80 single-stained, and CD206 single-stained controls.

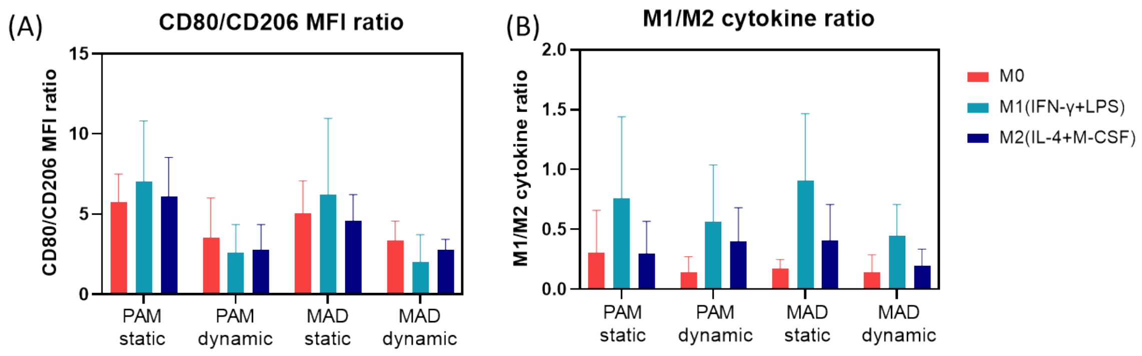

Figure S4. Characterization of polarized macrophages in bulk. (A) flow cytometry results and (B) ELISA results being compared for M1 and M2 markers.

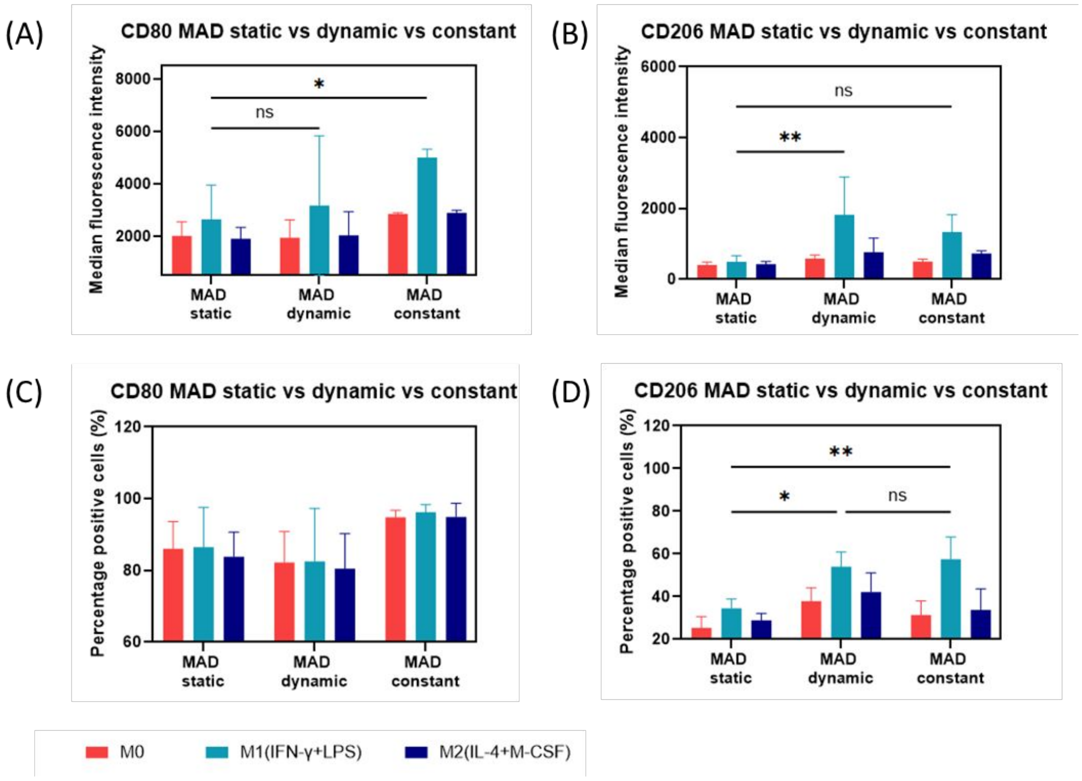

Figure S5. Flow cytometry results of median fluorescence intensity ((A) and (B)) and percentage positive cells ((C) and (D)) indicating CD80 and CD206 expression level among M0, M1 and M2-activated macrophages cultured on $_{\text{Mad}}$Surface in static, dynamic and constant condition. Values shown are mean values ± SD (n = 3). *Refers to P<0.05 and **refers to P<0.001.

Table S6 Cell number collected from hydrogel surfaces after detachment

Surface Types	M0			M1			M2		
PAM-static	3.57	2.16	3.18	3.21	1.56	2.67	3.57	2.22	4.5
PAM-dynamic	2.73	3.33	3.1	2.04	1.71	1.41	2.43	2.79	3.46
MAD-static	2.67	3.42	3.7	3.21	3.6	1.72	3.6	2.97	3.69
MAD-dynamic	2.55	3.78	2.89	1.65	2.25	1.36	2.46	4.05	2.76
